# A Noninvasive Score Model for Prediction of NASH in Patients with Chronic Hepatitis B and Nonalcoholic Fatty Liver Disease

**DOI:** 10.1155/2017/8793278

**Published:** 2017-03-02

**Authors:** Jing Liang, Fang Liu, Fengmei Wang, Tao Han, Li Jing, Zhe Ma, Yingtang Gao

**Affiliations:** ^1^Department of Gastroenterology and Hepatology, Tianjin Third Central Hospital, Tianjin 300170, China; ^2^Tianjin Key Laboratory of Artificial Cell, Tianjin 300170, China; ^3^Artificial Cell Engineering Technology Research Center of Public Health Ministry, Tianjin 300170, China; ^4^Molecular Biology Laboratory, Tianjin Third Central Hospital, Tianjin 300170, China; ^5^Department of Pathology, Tianjin Third Central Hospital, Tianjin 300170, China

## Abstract

*Aims*. To develop a noninvasive score model to predict NASH in patients with combined CHB and NAFLD.* Objective and Methods*. 65 CHB patients with NAFLD were divided into NASH group (34 patients) and non-NASH group (31 patients) according to the NAS score. Biochemical indexes, liver stiffness, and Controlled Attenuation Parameter (CAP) were determined. Data in the two groups were compared and subjected to multivariate analysis, to establish a score model for the prediction of NASH.* Results*. In the NASH group, ALT, TG, fasting blood glucose (FBG), M30 CK-18, CAP, and HBeAg positive ratio were significantly higher than in the non-NASH group (*P* < 0.05). Multivariate analysis showed that CK-18 M30, CAP, FBG, and HBVDNA level were independent predictors of NASH. Therefore, a new model combining CK18 M30, CAP, FBG, and HBVDNA level was established using logistic regression. The AUROC curve predicting NASH was 0.961 (95% CI: 0.920–1.00, cutoff value is 0.218), with a sensitivity of 100% and specificity of 80.6%.* Conclusion*. A noninvasive score model might be considered for the prediction of NASH in patients with CHB combined with NAFLD.

## 1. Introduction

Chronic hepatitis B (CHB) is prevalent worldwide, especially in Asia and Africa, with a high prevalence of 70% [1]. In China, it was estimated that at least 10% of the population was infected with hepatitis B, which is the most common chronic liver disease [2]. Currently, with the incidence of hyperlipidemia, obesity, and diabetes mellitus increasing significantly, the prevalence of nonalcoholic fatty liver disease (NAFLD) is on the rise in developed and developing countries, together with the incidence of nonviral cirrhosis and hepatocellular carcinoma [3]. Currently, the number of cases with concomitant CHB and NAFLD is increasing gradually. Previously, studies reported that the frequency of hepatic steatosis in CHB patients ranged from 27% to 51% [4]. The prevalence of biopsy-proven NAFLD was approximately 20% that of the CHB patients [5]. Therefore, the relationship and interaction between the two diseases is the focus of increased research attention.

NAFLD includes different degrees of liver damage ranging from simple fatty liver, nonalcoholic steatohepatitis (NASH), and fibrosis. Within the spectrum of NAFLD, simple fatty liver often remains stable for several years and might be associated with a low probability of progressive liver disease. However, NASH is more progressive and includes features of steatosis with hepatocyte injury, lobular inflammation, and fibrosis [6, 7]. Evidence suggested a possible increase in the risk of liver cirrhosis and hepatocellular carcinoma [8].

Both active hepatitis B and NASH contribute to increased alanine aminotransaminase (ALT) levels. Therefore, in patients diagnosed with both diseases, clinical assessment is important to determine the cause of the raised ALT level. Previous studies indicated that ALT elevation caused by hepatic steatosis masks real changes in ALT triggered by hepatitis B viral activation [9]. Thus, chronic hepatitis B patients might be easily misdiagnosed and treated with antivirals. Therefore, accurate diagnosis is essential to distinguish liver cell inflammation for appropriate intervention.

Although the relationship between hepatitis B virus infection and fatty liver is still not completely clear, lower viral response rates to interferon antiviral therapy were observed in patients with CHB and liver steatosis [10]. In the study by Jin et al., hepatic steatosis was significantly associated with entecavir failure in CHB patients [9]. The presence of steatohepatitis may affect the efficacy of antiviral therapy. Therefore, specific treatment strategies for steatohepatitis are needed to contribute to antiviral efficacy of chronic hepatitis B patients

It is necessary to distinguish NASH in patients with CHB combined with NAFLD for optimization of antiviral therapy and prevention of disease progression. Liver biopsy is still the gold standard for determination of disease status and definition of NASH. However, clinical application of liver biopsy is still limited because of the cost and invasive local sampling [11]. Hence, the purpose of this study was to develop a simple noninvasive scoring model to distinguish NASH in patients with combined CHB and NAFLD.

## 2. Materials and Methods

### 2.1. Study Population

The study was approved by the Institutional Review Board of Tianjin Third Central Hospital in 2013. Written informed consent was obtained from each patient for the use of their blood, liver biopsy, and clinical information.

A total of 65 patients with CHB and NAFLD treated at the Tianjin Third Central Hospital were included in this study from September 2013 to June 2015. All the patients were diagnosed according to the CHB diagnostic criteria recommended by the liver disease guidelines of the American Liver Disease Committee (2009) [12] and the NAFLD guidelines of the Japanese Liver Association (2015) [13]. The study subjects included 50 males and 15 females, with an average age of 39.3. The patients with combined CHB and NAFLD were diagnosed with steatosis by abdomen B ultrasound and liver biopsy. Exclusion criteria included (1) alcoholic fatty liver disease (alcohol intake exceeding 40 g/d in males and 20 g/d in females, over the past 5 years); (2) concomitant hepatitis B and hepatitis C; (3) autoimmune hepatitis or primary biliary cholangitis; (4) drug-induced hepatitis or toxic hepatitis; (5) genetic and metabolic liver diseases; and (6) liver carcinoma or biliary tract malignant tumor. All the patients signed informed consent before participation and liver biopsy. The patients were divided into 2 groups: NASH group (34 cases) and non-NASH group (31 cases) according to the liver pathological NAS score.

### 2.2. Laboratory Analysis of Serum Sample

Fasting venous blood was sampled one day before liver biopsy. Serum CK-18 M30 concentration was detected by enzyme-linked immunosorbent assay (ELISA, PEVIVA, Bromma, Sweden) according to the manufacturer's instructions. Biochemistry analysis of HBV markers included alanine aminotransferase (ALT), aspartic transaminase (AST), r-glutamine transpeptidases (r-GT), total bilirubin (TBil), triglyceride (TG), total cholesterol (TC), and fasting blood-glucose (FBG) levels. Serum HBVDNA was tested by real-time polymerase chain reaction (q-PCR).

### 2.3. Liver Stiffness and CAP Measurements

Liver stiffness measurement (LSM) and Controlled Attenuation Parameter (CAP) were measured 1–3 days before liver biopsy using FibroScan (Echosens, France). All the patients were analyzed with the 3.5 MHz M probe by an experienced operator who was blinded to the patient's diagnosis and data. Only results with 10 valid shots and interquartile range IQR/median liver stiffness ratio < 30% were considered reliable. Both LSM and CAP were obtained in the same area of liver parenchyma (between 25 and 65 mm in depth) [14]. The final LSM values and CAP values were expressed in Kpa and dBm^−1^, respectively.

### 2.4. Liver Biopsy and NAS Score

**Figure 1:**
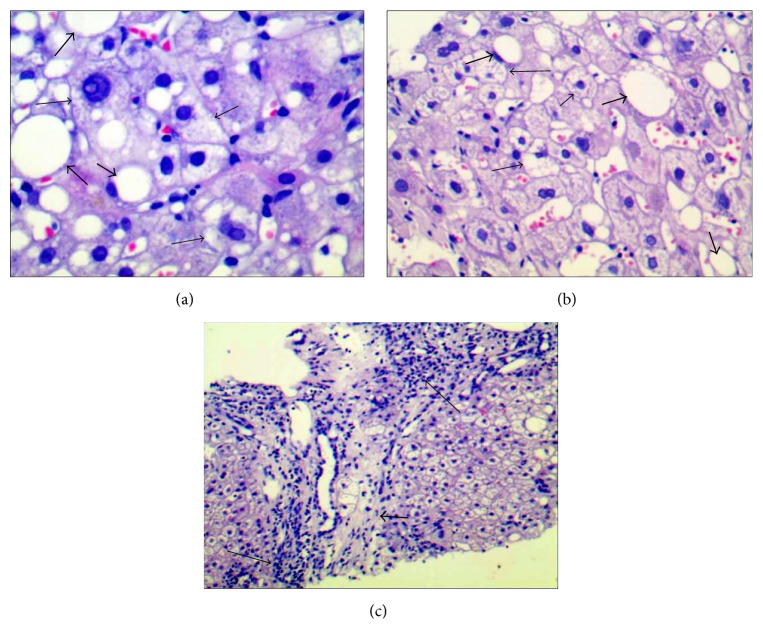
Characteristics of NASH in H&E-stained liver sections; fine arrows show clear hepatocyte ballooning and thick arrows show steatosis (haematoxylin-eosin stain, (a) original magnification ×400; (b) original magnification ×200); (c) illustrates lobular inflammation (fine arrows) and fibrosis (thick arrow) (haematoxylin-eosin stain, original magnification ×100).

Liver biopsy was performed by senior operators undergoing B mode ultrasonography using a 16 g bard disposable biopsy needle. Specimens were fixed with 10% polyformaldehyde solution and prepared as paraffin sections and stained with hematoxylin and eosin (HE). HE-stained liver biopsy samples were examined by two pathologists. The histological features of the liver were determined according to NAFLD histologic activity score (NAS) system [15]. The system identified the degree of steatosis (0 ≤ 5%; 1 = 5–33%; 2 = 34%–66%; 3 ≥ 66%), lobular inflammation (0: no foci, 1 < 2 foci per 200x field, 2: 2 to 4 foci per 200x field, and 3: foci per 200x field), hepatocyte ballooning (0: none; 1: rare or few; 2: many), and fibrosis score (0: no fibrosis, 1: perisinusoidal or periportal fibrosis, 2: perisinusoidal and portal/perioral fibrosis, 3: bridging fibrosis, and 4: cirrhosis). In our study, most cases were mild to moderate fibrosis (F0 : F1 : F2 : F3 = 5 : 13 : 39 : 8); the pattern of hepatic fibrosis was perisinusoidal mainly around the central vein. The NAS score including the sum of the above numerical pathologic scores and NAS scores ≥ 5 were considered as NASH (Figure 1).

### 2.5. Statistical Analysis

Statistical software SPSS 22 (SPSS Inc. Chicago. IL, USA) was used to analyze the data. Data were expressed as mean ± SD, and HBVDNA was calculated by denary logarithm. Intergroup comparisons were carried out using *t*-test. Comparison of data variables between the groups was carried out by chi-square test. Logistic regression analysis was performed to analyze the variables independently associated with NASH in the study population. The receiver operating characteristic (ROC) curve and the area under ROC (AUROC) curve were used to assess the parameters for NASH diagnosis. *P* < 0.05 was considered as statistically significant for all the analyses.

## 3. Results

### 3.1. Comparison of General Information

The study population was divided according to the NAS score, into NASH and non-NASH groups. No significant differences in clinical characteristics of age, gender, AST, rGT, TBIL, total cholesterol, HBVDNA level, and liver stiffness were found between the two groups. However, higher level of ALT, TG, FBG, and CK-18 M30 and CAP were observed in NASH compared with non-NASH group (*P* < 0.05), and the number of E antigen positive cases in NASH was fewer than that of non-NASH group (*P* < 0.05) (Table 1).

### 3.2. Univariate Analysis

ALT, TG, FBG, CK-18 M30, and CAP were analyzed by logistic regression analysis. Because E antigen positive cases were more frequent in NASH group than non-NASH group, we add HBVDNA level to logistic regression analysis. Multivariate analysis revealed that only CK-18 M30, FBG, CAP, and HBVDNA level were independently associated with NASH in CHB combined with NAFLD patients (Table 2).

### 3.3. Model to Predict NASH in Patients with CHB Combined with NAFLD

**Figure 2:**
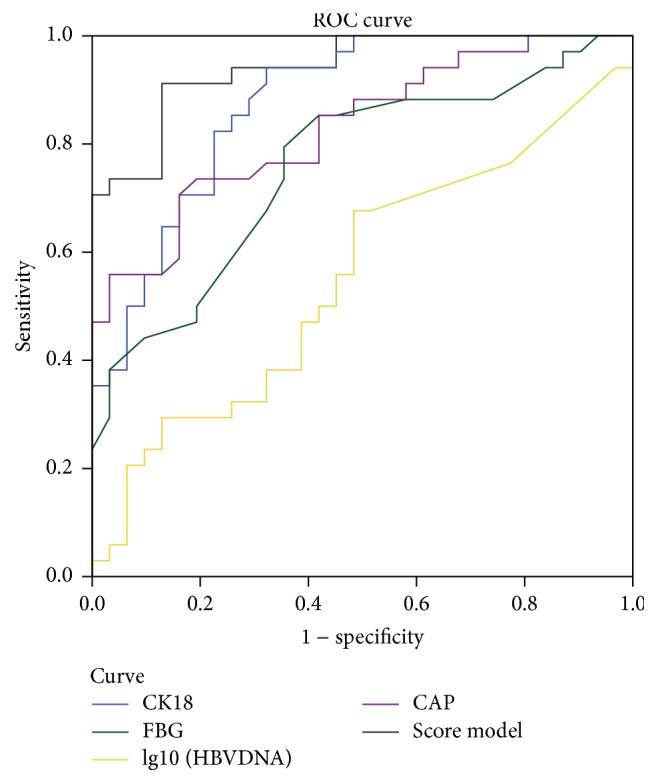
Model ROC curve for definitive NASH diagnosis in CHB combined with NAFLD.

The AUROC curves of CK-18 M30, FBG CAP, and lg10(HBVDNA) were 0.876 (95% CI: 0.794–0.959), 0.759 (95% CI: 0.642–0.876), 0.830 (95% CI: 0.733–0.927), and 0.553 (95% CI: 0.412–0.694), respectively (Table 3). A new model combining CK-18 M30, FBG, CAP, and HBVDNA was established through logistic regression. The equation of this model was −24.703 + 0.012 *∗* CK-18 M30 (U/L) + 2.032 *∗* FBG (mmol/L) + 0.045 *∗* CAP (dBm^−1^) − 0.564 *∗* lg10(HBVDNA). The AUROC curve for the prediction of NASH was 0.961 (95% CI: 0.920–1.000). A cutoff value was 0.218, with a sensitivity of 100% and a specificity of 80.6% (Figure 2).

## 4. Discussion

Chronic hepatitis B is a very common chronic liver disease. With the increasing incidence of fatty liver, it is easy to observe the coexistence of CHB and NAFLD clinically [16]. Studies suggested that concomitant hepatic steatosis is unlikely to have negative consequences for CHB [17]. However, other studies suggested that NAFLD was associated with an increased risk of cirrhosis and hepatocellular carcinoma in CHB patients [18, 19]. NAFLD encompasses a broad spectrum of clinical and histological manifestations, including hepatic steatosis, in which fat accumulation occurs without liver injury. NASH is characterized by progressive liver inflammation and varying degrees of fibrosis. Previous studies showed that NASH contributed to liver inflammation and disease progression [20]. Therefore, CHB patients with different levels of NAFLD show different clinical manifestations. It is therefore critical to identify NASH to identify the real cause of hepatocyte injury and control disease progression promptly and effectively.

Increased ALT levels are observed in both hepatitis B and NASH. In a study of patients with both fatty liver and hepatitis B by Spradling [21], a raised ALT level was attributed to steatohepatitis in patients who were HBeAg negative. However, in HBVDNA-positive patients, a raised ALT should be used to distinguish hepatitis B from NASH [22]. The Asia-Pacific Association for the Study of liver (APASL) guideline [23] recommends ALT and HBVDNA levels as indicators of active hepatitis B. However, the guideline does not mention the clinical conditions in which CHB and NASH coexist. A raised ALT level due to NASH could interfere with the treatment of hepatitis B. Therefore, ALT level is not appropriate to distinguish hepatitis B from NASH. Furthermore, in our study, a higher ALT level was observed in CHB with NASH groups than in CHB with non-NASH group. However, it was not an independent factor for the presence of NASH in logistic regression analysis. Therefore, further studies are needed to determine whether a differential diagnosis of NASH before antiviral therapy or adjusting the antiviral treatment for CHB combined with NAFLD was the most appropriate.

Conventional imaging techniques such as ultrasonography, CT, and MRI can be used to detect hepatic steatosis and fibrosis. However, their clinical utility was limited by the operator skill and technical limitations of ultrasonography, CT radiation, and high cost of MRI. Furthermore, these techniques do not facilitate the diagnosis of NASH in patients with CHB combined with NAFLD. Liver biopsy is considered the gold standard in assessing hepatic pathology in chronic liver disease. However, its acceptance rate was still low in clinical practice. Therefore, it is essential to develop a method to distinguish NASH in patients with combined CHB and NAFLD.

Our study assessed several variables to distinguish NASH from patients with CHB plus NAFLD. Cytokeratin 18 (CK-18) is a major intermediate filament protein associated with the structural changes characteristic of apoptosis in the liver [24]. During the course of NASH, apoptosis was the most obvious physiological manifestation [3]. Increasing evidence suggested that CK-18 fragment M30 was closely related to hepatocyte inflammation and NASH [25]. Furthermore, CK-18 M30 was correlated with apoptosis and NAS score, which is the gold standard of diagnosis of NASH [15]. In our previous study [26], CK-18 M30 was significantly increased in patients manifesting CHB with NAFLD and was positively correlated with ALT, TG, FBG, histology inflammation score, fibrosis score, and steatosis. In this study, serum CK-18 M30 level was significantly higher in NASH group than in non-NASH group and was an independent variable in the logistic regression analysis of patients manifesting CHB combined with NASH. CK-18 M30 may reflect the presence of NASH in liver cell injury and apoptosis.

Liver stiffness is a noninvasive marker of fatty liver and hepatitis B fibrosis [27]. However, it could interfere with the transmission of shear waves in obese patients. Nonetheless, liver stiffness may reflect the different stages of liver fibrosis. However, studies evaluating liver steatosis are rare. Our study also found no significant difference in liver stiffness between NASH and non-NASH groups, while the CAP index was significantly increased in the NASH group. Logistic regression revealed that only CAP was an independent predictor of NASH in patients diagnosed with combined CHB and NAFLD. Currently, CAP is used to detect steatosis and for semiquantitative evaluation of fatty content according to the principle of instantaneous elastic wave vibration [28]. Studies suggested that it facilitated accurate determination of steatosis associated with fatty liver, hepatitis B, hepatitis C, and other chronic liver diseases [29, 30]. Furthermore, the efficacy of CAP for detection of steatosis was 10% [31], which is more sensitive compared with other imaging techniques. In our study, the area under the ROC curve of CAP for detecting NASH was 0.830, suggesting that CAP enabled accurate evaluation of NASH based on fat content in patients with concomitant CHB and NAFLD.

Recent studies have focused on the causes of steatosis in CHB patients. Kim et al. showed that increased HBV X protein expression induced lipid accumulation in hepatocytes [32], while other studies reported that insulin resistance and metabolic disorders were the main factors underlying the pathogenesis of steatosis [33]. Our study found that TG and FBG were significantly higher in the NASH group than in the non-NASH group, although only FBG was an independent predictor for the presence of NASH. Hyperinsulinemia and hyperglycemia were observed not only in obese patients but also in nonobese, nondiabetic patients with NASH, and insulin resistance and diabetes mellitus were associated with the development of NASH [34, 35]. Continuous glucose monitoring revealed that early glucose intolerance was an indicator of the severity of hepatic fibrosis in children with NAFLD [36]. Glycemic variability is an independent factor predicting impaired glucose metabolism and indicated the progression and fibrosis of NAFLD [37]. Our study provides additional evidence supporting the role of fasting blood glucose in metabolic abnormalities of patients diagnosed with CHB combined with NAFLD.

We found that, in patients with combined CHB and NAFLD diagnosed with B ultrasound, the NAS score of NASH patients accounted for 52.3%. Therefore, disease status and the presence of NASH in patients with combined CHB and NAFLD were not adequately diagnosed with B ultrasound alone. Logistic regression analysis revealed CK-18 M30, CAP, and FBG as independent predictive factors of NASH in patients with combined CHB and NAFLD, associated with apoptosis, fat content, and metabolic abnormality of NASH, respectively. These three indicators well distinguished NASH from NAFLD; however, there were inadequate to discriminate from NASH and CHB. In our study, we also found HBVDNA level was negatively associated with NASH in patients with combined CHB and NAFLD. Therefore HBVDNA level was also the important factor for the identification between active hepatitis B and NASH. According to the ROC curve, the sensitivities of CK-18 M30, CAP, FBG, and lg10(HBVDNA) were 94.1%, 76.5%, 79.4%, and 67.6%, and the specificities were 67.7%, 87.1%, 64.5%, and 51.6%, respectively. When combined with the above three factors, the sensitivity and specificity of the diagnosis of NASH were 100% and 80.6%. Therefore, a new model that combined CK-18 M30, CAP, FBG, and HBVDNA level based on logistic regression might represent a simple, noninvasive, and convenient method to predict NASH in patients with CHB plus NAFLD. Determination of NASH using this method facilitates screening of patients for lifestyle intervention, control of hepatocyte inflammation for prevention of disease progression, and determination of the etiology of elevated ALT for accurate antiviral strategy.

The limitations of the study include small sample size of liver biopsy specimens, involving patients at a single center. Large-scale, multicenter cohort studies are needed for further investigation of patients manifesting CHB combined with NAFLD.

## 5. Conclusion

In conclusion, this study established a new noninvasive score model, which included CK-18 M30, CAP, FBG, and HBVDNA to diagnose NASH in patients with combined CHB and NAFLD. Its clinical significance relates to assessment of disease progression and anti-hepatitis B management.

## Figures and Tables

**Table 1:** Clinical and serological characteristics of the study population.

Factor	All subjects (*n* = 65)	Non-NASH (*n* = 31)	NASH (*n* = 34)	*P* value
Age (years)	39.80 ± 11.21	38.00 ± 10.56	41.4 ± 11.68	0.219
Gender (M/F)	50/15	24/7	26/8	0.928
ALT (u/L)	73.24 ± 62.50	55.52 ± 49.49	89.41 ± 69.17	0.028^*∗*^
AST (u/L)	29.86 ± 27.47	26.71 ± 30.34	32.74 ± 24.69	0.381
GGT (iu/L)	42.35 ± 20.71	37.64 ± 18.21	46.64 ± 21.15	0.080
Tbil (umol/L)	14.92 ± 6.05	14.06 ± 5.50	15.71 ± 6.50	0.275
TC (mmol/L)	4.35 ± 1.03	4.25 ± 0.99	4.45 ± 1.07	0.437
TG (mmol/L)	1.71 ± 0.55	1.53 ± 0.44	1.87 ± 0.88	0.008^*∗∗*^
FBG (mmol/L)	5.19 ± 0.81	4.80 ± 0.64	5.54 ± 0.79	0.000^*∗∗*^
Lg10 (HBVDNA)	5.00 ± 2.16	4.75 ± 2.12	5.23 ± 2.20	0.371
HBeAg positive (*n*)	25	16	9	0.037^*∗*^
CK-18 M30 (u/L)	569.60 + 429.54	345.13 ± 136.13	774.26 ± 500.49	0.000^*∗∗*^
LSM (Kpa)	8.62 ± 4.59	7.63 ± 4.16	9.53 ± 4.83	0.095
CAP (dBm^−1^)	270.92 ± 45.70	243.10 ± 31.12	296.28 ± 42.19	0.000^*∗∗*^

^*∗*^ *P* < 0.05, ^*∗∗*^*P* < 0.01.

*n*, number; M, male; F, female; *P* value corresponds to the comparison of the two groups. ALT, alanine aminotransferase; AST, aspartic transaminase; r-GT, r-glutamine transpeptidases; TBil, total bilirubin; TG, triglyceride; TC, total cholesterol; FBG, fasting blood-glucose; LSM, liver stiffness measurement; CAP, controlled attenuation parameter.

**Table 2:** Multivariate analysis of elevated variables.

Factor	*P* value	OR	OR 95% CI
ALT (u/L)	0.659	0.997	0.982–1.012
TG (mmol/L)	0.908	1.109	0.192–6.405
FBG (mmol/L)	0.007^*∗∗*^	7.632	1.749–33.304
CK-18 M30 (u/L)	0.010^*∗*^	1.012	1.003–1.021
CAP (dBm^−1^)	0.011^*∗*^	1.046	1.010–1.084
lg10(HBVDNA)	0.042^*∗*^	0.569	0.331–0.979

^*∗*^ *P* < 0.05, ^*∗∗*^*P* < 0.01.

OR, odds ratio; 95% CI, confidence interval.

**Table 3:** ROC curve of the predictive factors and score model.

Factor	AUROC	AUROC 95% CI	Sensitivity	Specificity	Cutoff value
FBG	0.759	0.642–0.876	0.794	0.645	4.95 (mmol/L)
CK-18 M30	0.876	0.794–0.959	0.941	0.677	394.50 (U/L)
CAP	0.830	0.733–0.927	0.765	0.677	265 (dBm^−1^)
lg10(HBVDNA)	0.553	0.412–0.694	0.676	0.516	3.54
Model	0.961	0.920–1.000	1.00	0.806	0.218

AUROC, area and the ROC curve.
